# Supplementary material for: Genome-wide analysis of a collective grave from Mentesh Tepe provides insight into the population structure of early neolithic population in the South Caucasus
Source: Commun Biol. 2023 Mar 25;6:319. doi: 10.1038/s42003-023-04681-w (PMC10039893; doi:10.1038/s42003-023-04681-w)
Supplement: Supplementary file 2 — Supplementary Information [file 42003_2023_4681_MOESM2_ESM.pdf]

Supplementary Information

for

Genome-wide analysis of a collective grave from Mentesh Tepe provides insight into the population structure of early neolithic population in the South Caucasus.

Perle Guarino-Vignon, Mael Lefeuvre, Amelie Chimenes, Aurore Monnereau, Farhad Guliyev, Laure Pecqueur, Elsa Jovenet, Bertille Lyonnet, Celine Bon

2023

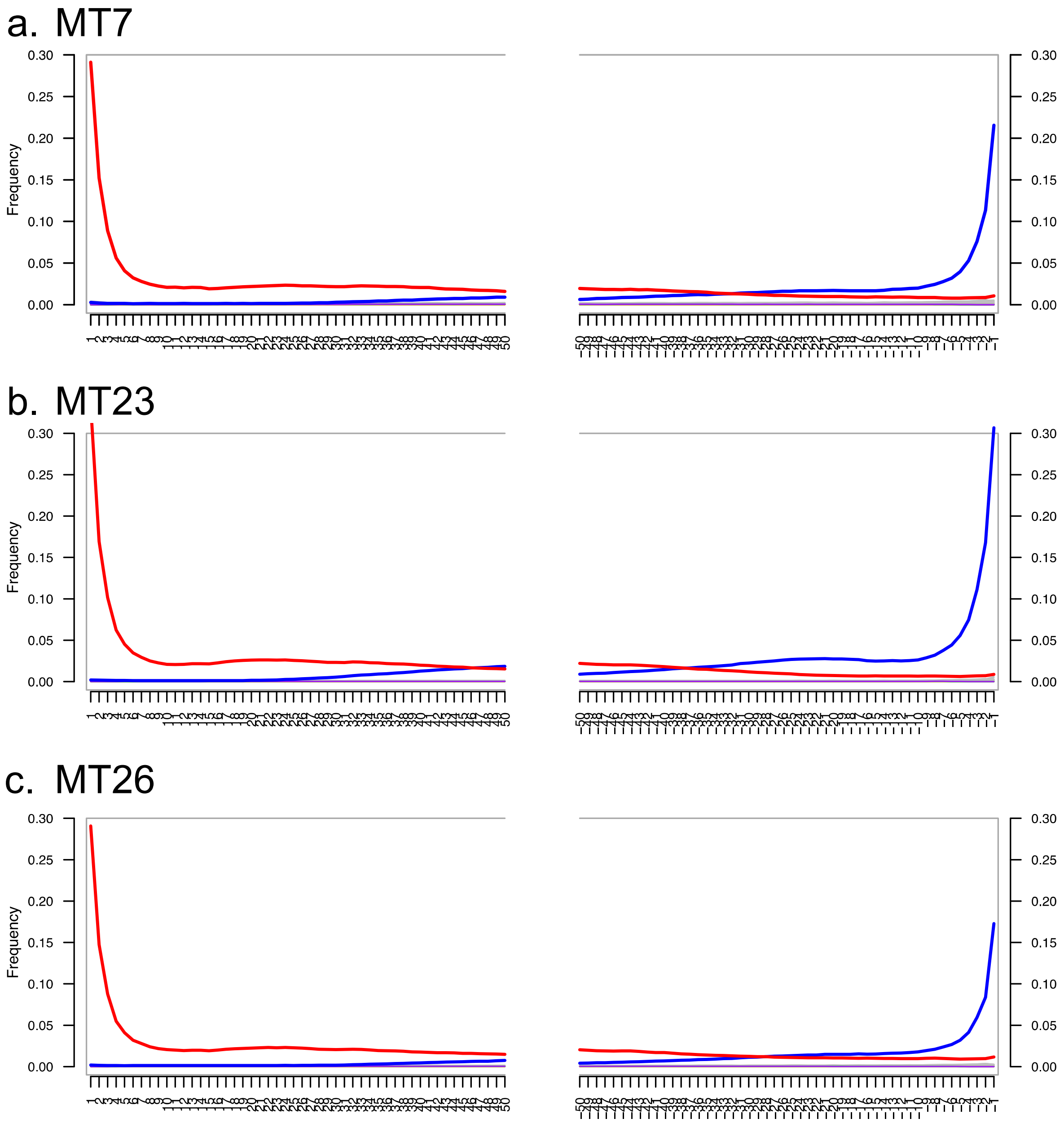

Supplementary Figure 1

DNA damage profile obtained using MapDamage v2 for (a) MT7, (b) MT23, (c) MT26.

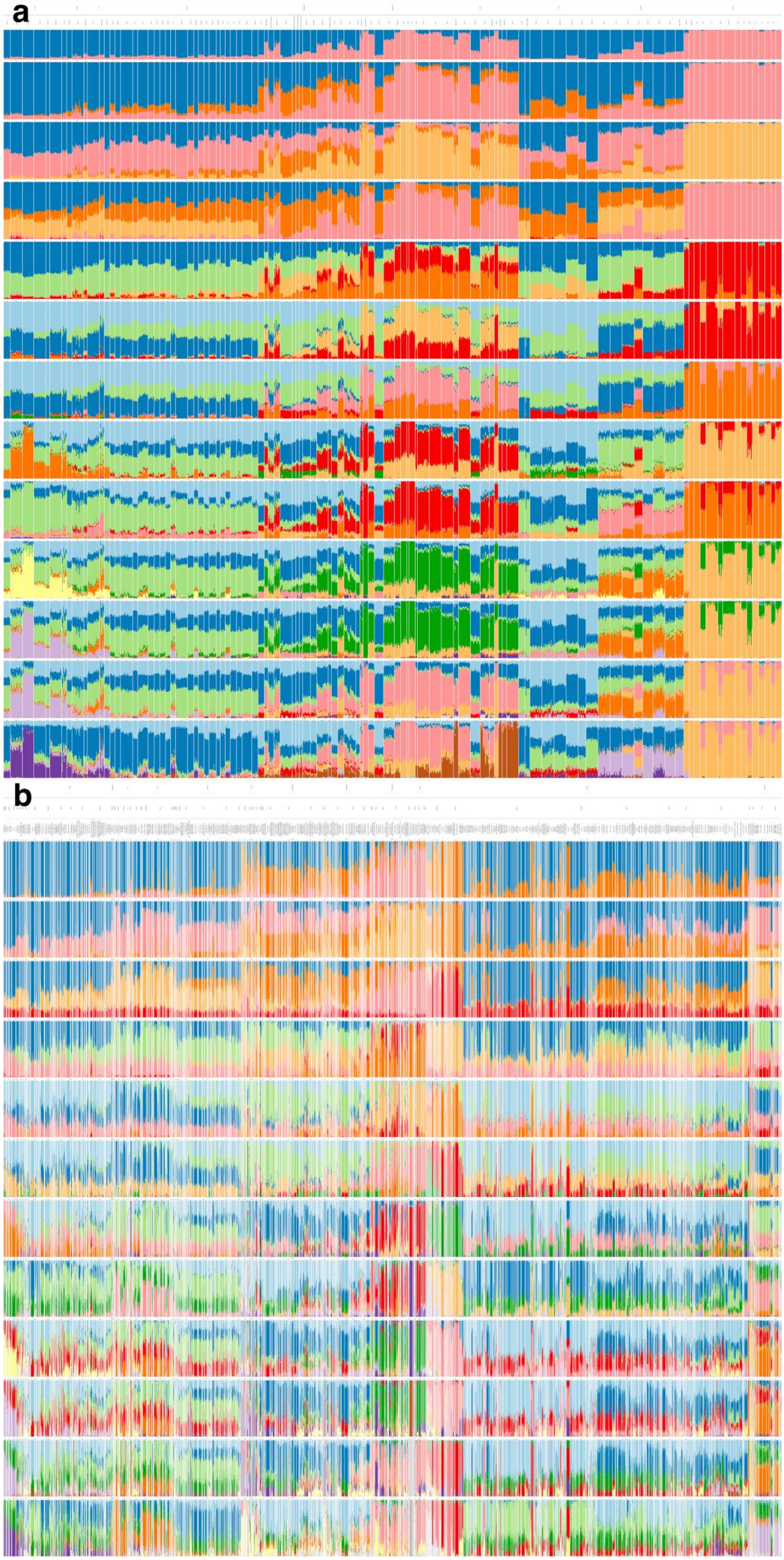

Supplementary Figure 2
Full ADMIXTURE analysis result for the modern (a) and ancient (b) individuals from the HO-dataset.
A bigger version of the admixture is available here: https://github.com/pguarinovignon/Mentesh/tree/main/2.PopGen%20Analysis/ADMIXTURE

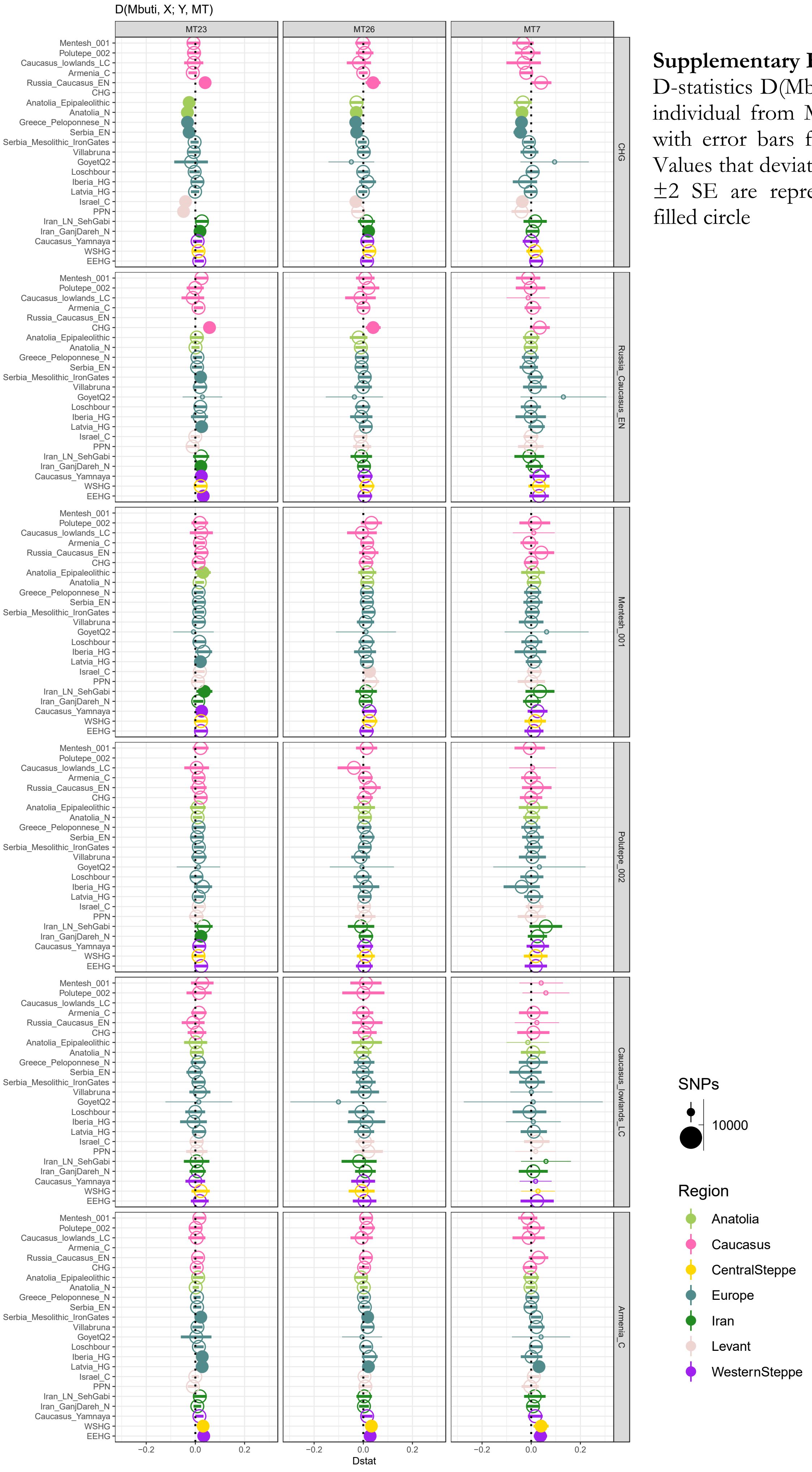

Supplementary Figure 3 D-statistics D(Mbuti, X; Y, each individual from Mentesh Tepe), with error bars for the ±2 SE. Values that deviate from 0 in the ±2 SE are represented with a filled circle

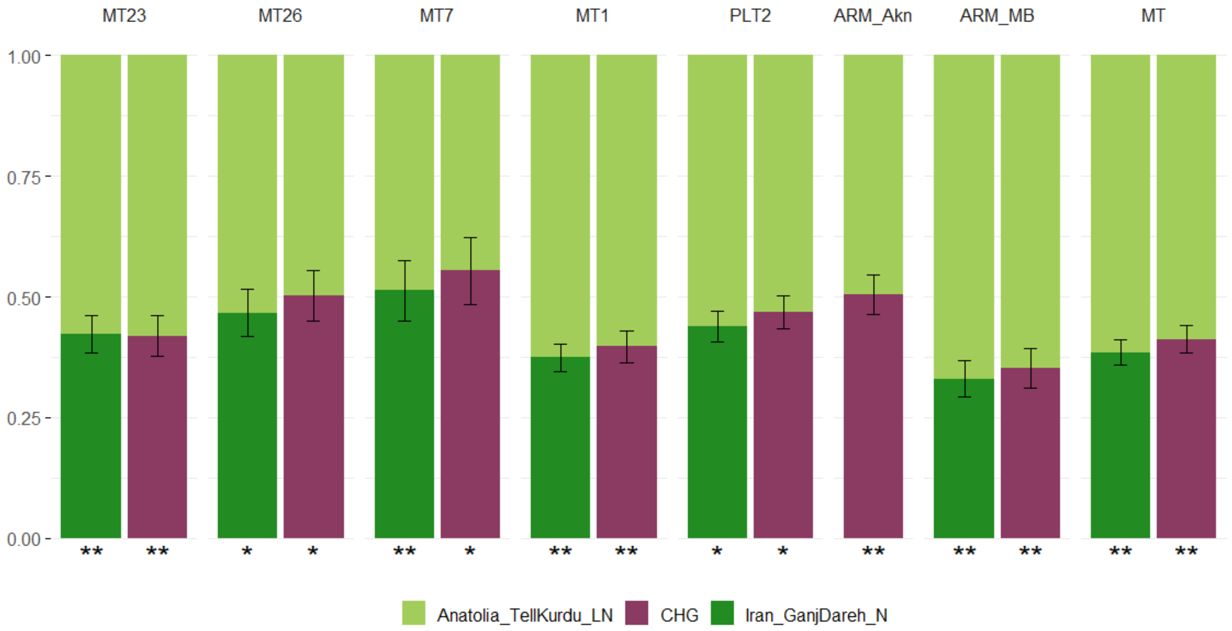

Supplementary Figure 4
Ancestry proportions of each Neolithic South-Caucasus individual on autosomes, calculated using qpAdm. Two-stars models indicate a p-value>0.05, one star models a p-value>0.01. Ancestry proportions are plotted with error bar representing ±1 SE.

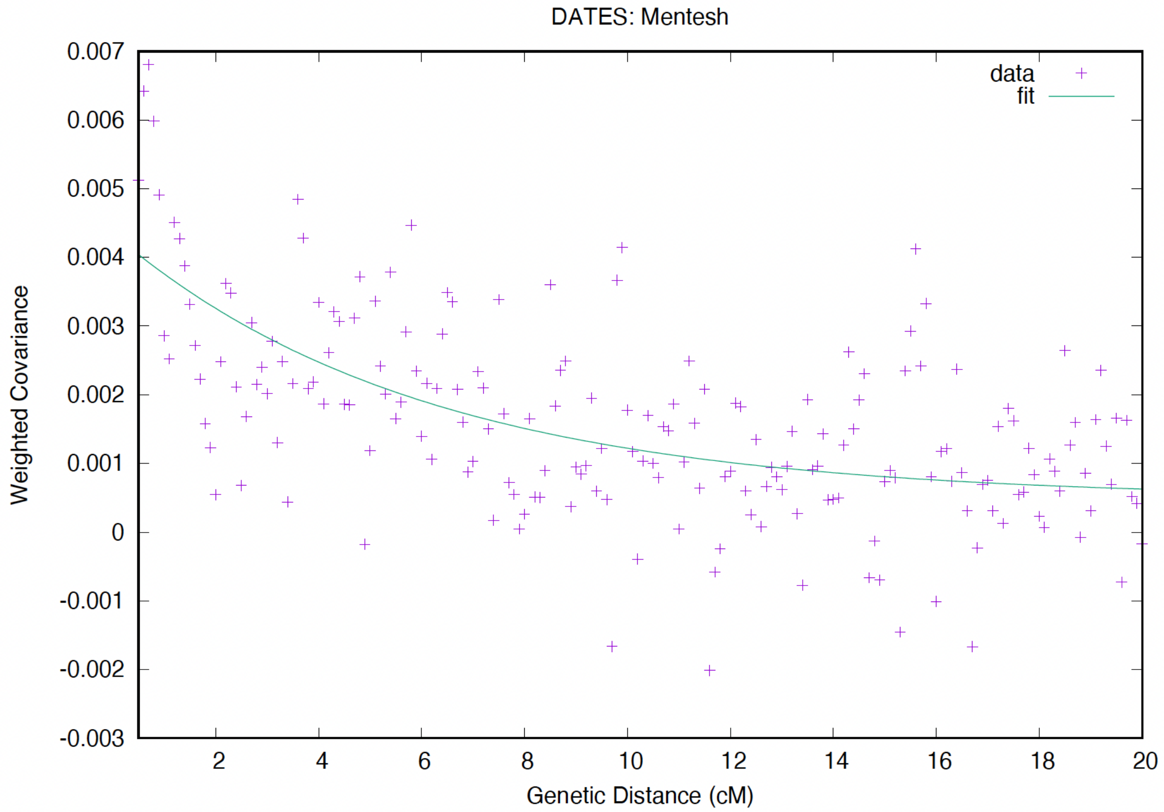

Supplementary Figure 5
Decay of ancestry covariance estimated by DATES for Mentesh Tepe individuals

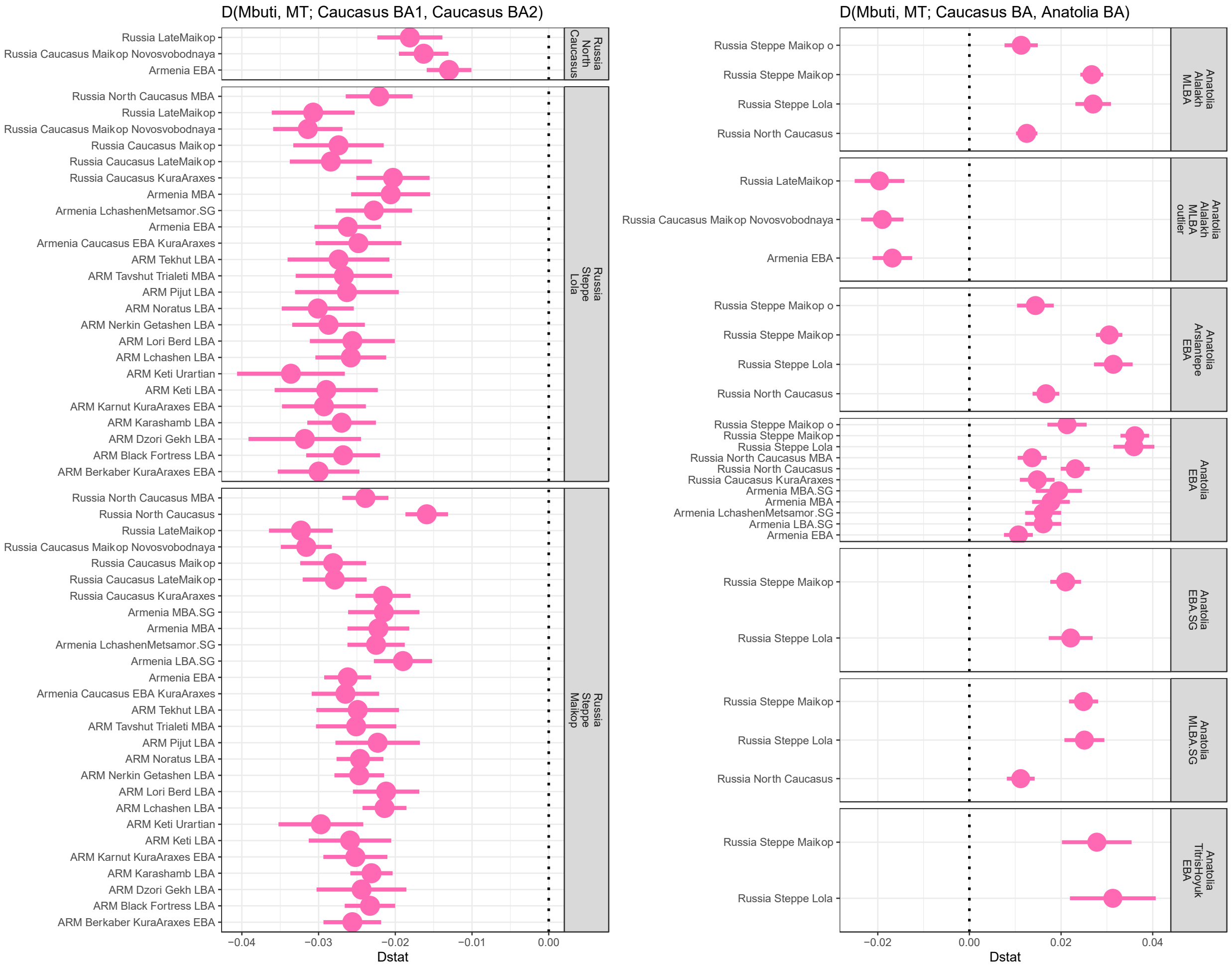

Supplementary Figure 6

D-statistics D(Mbuti, MT; Caucasus BA1, Caucasus BA2) and D-statistics D(Mbuti, MT; Caucasus BA, Anatolian BA), where Caucasus BA and Anatolian BA represent different populations from the Bronze Age Caucasus and Anatolia, with error bars for the ±2 SE. Only values that deviate from 0 in the ±2 SE are represented.

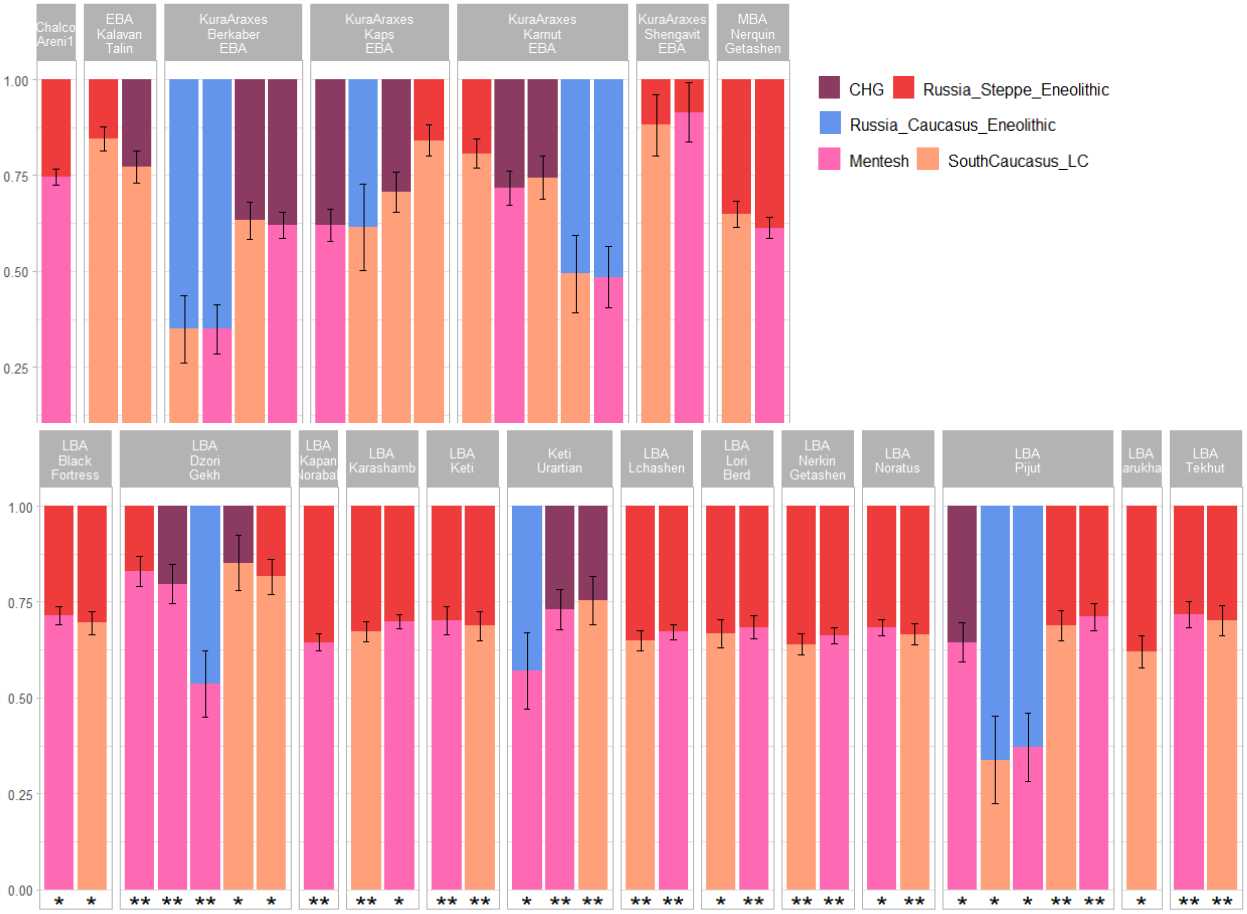

Supplementary Figure 7

Ancestry proportions of Bronze Age South-Caucasus populations on autosomes, calculated using qpAdm. Two-stars models indicate a p-value>0.05, one star models a p-value>0.01. Ancestry proportions are plotted with error bar representing ±1 SE.

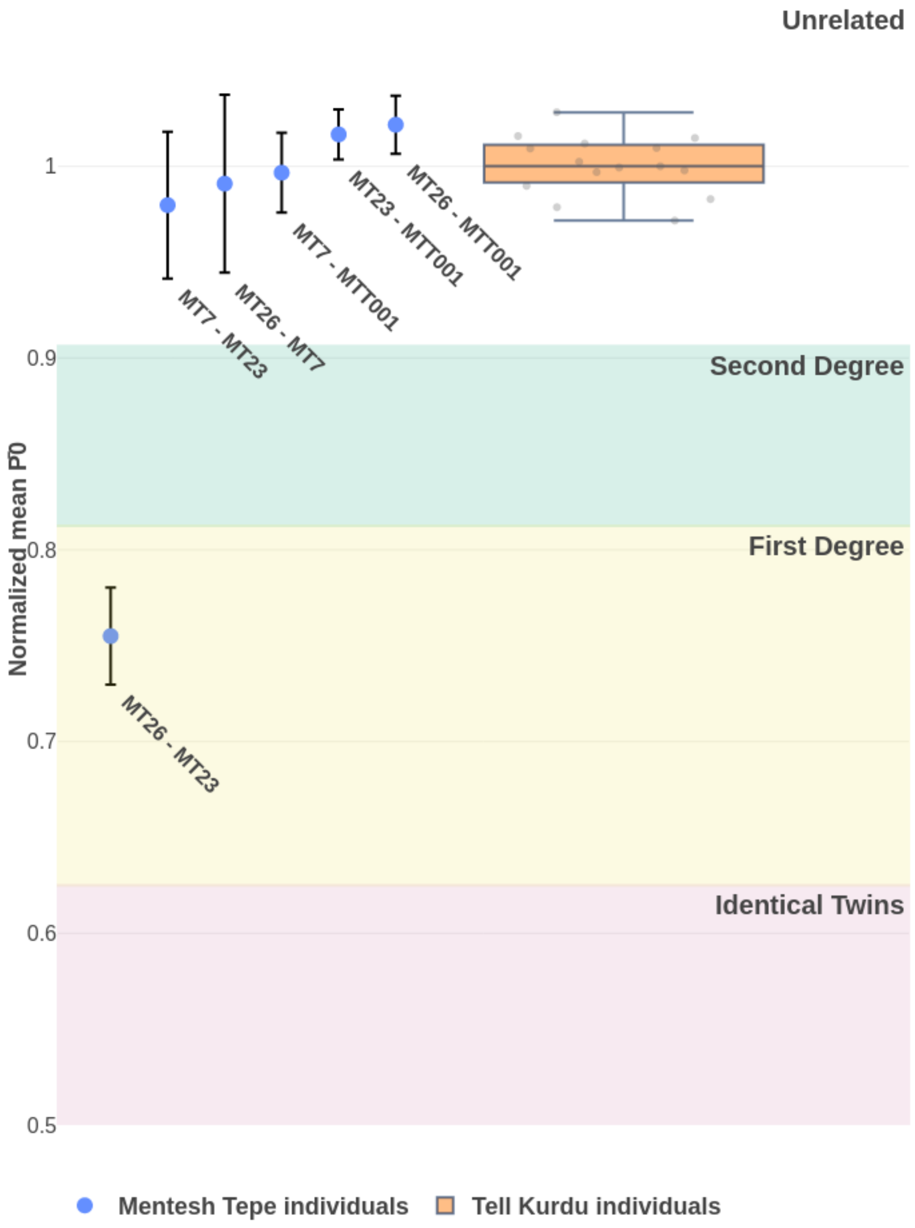

Supplementary Figure 8

Sorted normalized average P0 values for all pairwise comparisons between Mentesh Tepe individuals (blue markers) and Tell Kurdu individuals (orange boxplot), using the READ estimation method. Marker error bars indicate 95% confidence intervals for the observed average P0 estimation. Colored areas represent the thresholds at which relatedness orders are considered by READ. Boxplot highlights the distribution of the observed normalized P0 of the Tell Kurdu individuals used for the normalization step of READ, with each point corresponding to a given pair of proxy individuals (jittered along the horizontal axis). Lower and upper fences respectively represent the minimum and maximum sample point, excluding observations located below or above 1.5 times the inter-quartile range. Boxspan: 25th – 75th percentile.

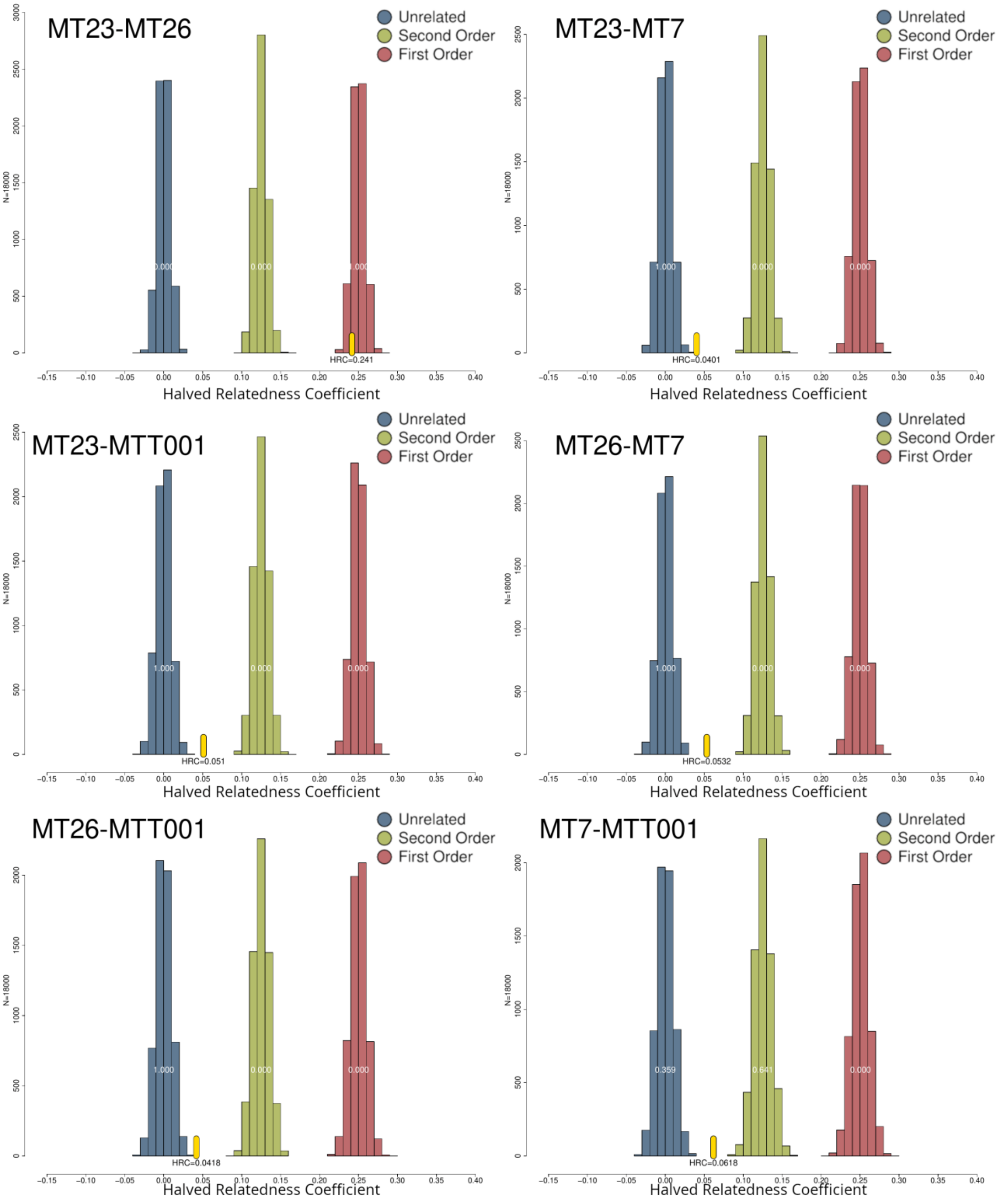

Supplementary Figure 9
Estimation of genetic relatedness of newly and previously sequenced Mentesh Tepe individuals with TKGWV2.

Supplementary Note 1:
Data concerning the archaeological sites mentioned in the text

Aknashen, Armenia
See below at Shomu-Shulaveri culture

Alkhantepe, Azerbaijan
Excavations directed by T. Akhundov (Institute of Archaeology and Ethnology, Baku) between 2008 and 2012.

The settlement (N 39°21'607"; E 48°27'720") is situated in the Mughan Plain, north of the village of Uchtepe in the Jalilabad district. It had no visible mound on the surface due to modern cultivation but its overall size is said to have covered over 4 ha of which 220m2 have been excavated. The site has 3m thick deposits divided in 7 construction levels all belonging to the same Late Chalcolithic period contemporary with the Leilatepe cultural horizon but an Iron Age settlement is contiguous on the south-west [1,2]. From a palynological analysis, the climate is said to have been more humid at that time than today, with forests around while the level of the Caspian Sea was higher and therefore the coast closer to the site. The settlement was built on the right bank of a water channel now not functioning anymore. The constructions have various plans. An earthquake is said to be responsible for the most important changes that happened after level 3. The earliest level 7 contained dug-out and semi dug-out small circular constructions (diameter 2,4m), levels 6 and 5 provided semi dug-out rectangular ones with mudbrick walls, level 4 gave above-ground rectangular and oval ones associated with circular and rectangular dug-out and semi dug-out buildings, and level 3 produced circular and oval semi dug-out constructions. In level 3 were found 8 graves (both adults and children) said to have been dug from level 2. In level 2 wattle and daub rectangular constructions have been brought to light, while level 1 consisted of a strange platform including partly burnt wooden beams put at right angles to make squares the function of which is unclear. In the different layers, several kilns for ceramic production and possibly also for metallurgy, have been found. Three burnt to red circular places ca. 1m in diameter, with a small container in the center are considered as altars while six circular platforms (ca. 0,5m in diameter) of function unknown are made of pottery sherds.

Fourteen graves with individuals of different ages from infants to adults have been discovered in the different layers, one of which is dated on a tooth 3776-3651cal BC (see [3]). The adults were in pits, children in jars. The abundant pottery, part of which was probably done on a slow wheel is typical of the Leilatepe culture, light colored, mostly vegetal tempered with round and flat bottoms and sometimes wearing incised potters' marks. Among the various shapes, recipients with a double opening are frequent. Some of the sherds wear traces of basketry or of cloth. A few fragments with painted decoration are also present as well as a peculiar and new black or dark grey ware often hightly polished in a clay without temper and always with flat bottoms. Also foreign to the Leilatepe culture are a few vases with a ring foot and one with a pedestal-base, as well as a number of handles often related to the black ware mentioned above. Crucibles and moulds for metallurgical production and numerous slags are present, but only a few metal implements were discovered. Anthropomorphic and zoomorphic figurines, and one stamp seal have also been found. The lithic industry is made of both flint and obsidian. The author dates the site to the middle of the second half of the 4th millennium due to the presence of the black ware that he sees as a forerunner of the KA pottery, but the date seems too late as shown by radiocarbon dates on several graves[3] (and Herrscher and Poulmarc'h personal communication).

One individual from Alkhantepe (ALX002) was analyzed for aDNA and is included in[3] genetic analyses.

Aratashen-Shulaveri-Shomu culture (South Caucasus),

See below at Shomu-Shulaveri culture

Areni 1, Armenia

Excavations directed by B. Gasparyan (Institute of Archaeology and Ethnography, National Academy of Sciences, Armenia) together with R. Pinhasi (School of Archaeology, University College , Dublin, Ireland) and G. Areshian (Cotsen Institute of Archaeology at UCLA, USA).

Areni-1 is a three-chambered cave located in the Vayots Dzor Province of southern Armenia. It stands on the left-hand side of the Arpa River basin, a tributary of the River Araxes. Late Chalcolithic and Early Bronze Age (4300–3800 BCE) as well as Medieval (14th c. CE) occupations have been exposed with perishable organic materials exceptionally well preserved. A unique charcoal but with no related material also shows a possible occupation during the Neolithic period. The Late Chalcolithic occupation is dated between 4300-3400 BCE by 8 radiocarbon dates. The cave was used for habitation, for keeping animals and storing plants, as well as for the production of wine, and for ritual purposes. The site also yielded secondary burials as shown by three subadult crania dated to the last third of the 5th millennium and deposited each in a clay pot sealed a few centuries later, during the first third of the 4th millennium. According to one article[4], the evidence from Areni-1 suggests that the Kura-Araxes culture developed from this Late Chalcolithic culture. This is drawn from the presence of some potteries with a black-burnished exterior, semi-globular handles and shapes typical of the KA cultural assemblage but usually dated not earlier than the second half of the 4th millennium. The authors consider however that this KA pottery started already at the very beginning of the 4th millennium, as there is no visible hiatus in the occupation. However, another article on the same site[5] gives a new set of radiocarbon dates and presents evidences of other occupations, and most importantly of one during the early phase of the KA period (six radiocarbon dates including on a leather shoe are place between ca. 3650 and 3350 if not 3000 BCE). An Early Iron Age level (9th-8th c. BC) that gave some pottery and a bronze adze is also mentioned while the medieval occupation is extended to the 8th-9th and 12th-14th centuries AD. In this second article, new pottery groups are added to those presented in the first article, but, unfortunately with no pictures or drawings. One of these new groups is said to be a well-fired, dark-grey burnished, thin-walled pottery related with the Iranian plateau (but it also recalls the pottery recently discovered at Alkhantepe in Azerbaijan). Another pottery group is considered as similar to the pre-Maikop pottery of the North-Caucasus sites like Zamok or Svobodnoe leading the authors to reconsider the Maikop chronology though this had already been done elsewhere [6,7]. Altogether, the cave has been occupied at several different periods not all identified at once so that one should take with caution the dates said to be “based on context” in [8] supplementary information: 7-8, and thus the conclusions reached on the DNA studies for these samples.

Five DNA individuals were published by[8], allegedly associated with the Chalcolithic: ARE12.1, ARE20, AR1/43C, AR1/46, AR1/44. In this publication only the last three were radiocarbon dated, the first two being dated through the archaeological context in which they were found. It seems however that ARE20 has later been radiocarbon dated on a tooth 4229-3985 calBCE (5260±30 BP, Poz-81110) (given in the metadata file from the Allen Ancient DNA Resource v.50). Thus, only ARE 12.1 is still dated through the archaeological context that, as shown above, is not totally secure.

Chokh, Daghestan

Excavations by different authors, the most recent ones directed by Kh. A. Amirkhanov (Institute of Archaeology, Moscow).

First explored at the end of the 1950's, the site has been re-excavated by Kh.A. Amirkhanov in the 1970s and 1980s and dated mainly to the Mesolithic and Neolithic periods, with a small Bronze Age level reoccupation [9]. The site is located on the Turchidag plateau of central Daghestan, between 1750 and 2240m asl. Two levels (E and D) are attributed to the Mesolithic and include specific tools like the "Chokh point" and various singular microliths. They are separated by a probably short hiatus from the next, Neolithic, level (C) (but see at the end of this short review a revision of the chronology). In this last level, the first grains of cereals (*Triticum monococcum*, *dicoccum*, *Hordeum vulgare*) have been brought to light attesting a production economy. The presence of a specific local cereal (*Triticum aestivo-compositum*) has been considered as an evidence of an autonomous domestication of the local plants. Domesticated animals (sheep and bovids), and often undertermined wild or domesticated goats are present. This Neolithic level also gave the first evidence of architecture in the shape of above-ground more or less circular large houses (ca. 60m2) made with stone or slabs and one with an entrance corridor. Several hearths at different levels and numerous pottery sherds were found. The roof of the houses was probably supported by a central pole as shown by the presence of a large stone at that place. The lithic industry is made of flint and though it shows evident genetic links with the previous Mesolithic layers as shown by the persistence of the Chokh points, new types of tools are introduced by then. Other stone tools in limestone and schist for grinding or hammering point towards the presence of activities related to a production economy. A number of bone tools have also been discovered. About 900 very fragmented handmade (coils or slabs) pottery sherds have been discovered especially around the fireplaces. The surface is smoothed inside and outside except that of the flat bottom. The clay was tempered mostly with mineral elements and most of the sherds have a dark core. The colors are irregular from yellowish to dark grey, rarely reddish. Only two sherds present an applied decoration, one made of two lumps reminding that frequent in the Shomu-Shulaveri culture, the other consisting in a band separated in two. This pottery finds no comparisons in other sites of Daghestan like that of Ginchi or of the Rugudzhin open-places and is closer to that of the Shomu-Shulaveri culture.

This description of the finds within the different layers has however to be taken with great caution as shown by the results recently published on the 23 radiocarbon samples analyzed in three different laboratories[10]. From there, it seems that most of the finds considered as Neolithic actually date to the Bronze Age (especially mid-second millennium cal BC), and that the Mesolithic occupation dates back mostly to the 11th millennium cal BC. However, two samples analysed at Kiev and at the RAN laboratory in Moscow do point at an occupation around 6000 BC, i.e. more or less contemporary with the Damjili Cave in Azerbaijan. Thus, level C is very heavily disturbed by the later C1/Bronze Age occupation.

Damjili Cave, Azerbaijan

Excavations directed by Y. Nishiaki in 2016-2017.

This rock shelter, 10km NW of Gazakh in Western Azerbaijan, is up to now the unique known missing link between the Mesolithic and Neolithic periods in southern Caucasus. Recent excavations by a Japanese-Azerbaijanese team[11] have revealed a superimposed accumulation of well-preserved cultural levels well dated with 20 radiocarbon dates from the Medieval (8th-10th c. CE) down to the Middle Paleolithic period, including small occupations during the Bronze Age (ca. 4800-4200 cal BP) and the Chalcolithic period (ca. 6500-5700 cal BP). Most important are the Neolithic levels = Unit 4 (ca. 7700-7300 cal. BP) and the Mesolithic ones = Unit 5 (ca. 8400-8000 cal. BP). The most obvious difference between units 4 and 5 is the total absence of pottery in unit 5, while that of unit 4 is typical of the Shomu-Shulaveri Culture in an already later stage with coarse plant-temper like that of Göytepe with which it is partly contemporary and may even have persisted later according to the 14C dates. The overall lithic assemblages of Units 4 and 5 is quite similar but differ remarkably from that of the

Neolithic site of Göytepe and even that of Hacı Elamxanlı Tepe, the earliest known site of this period with Aknashen in Armenia. Nevertheless, Units 4 and 5 display dissimilarities in many ways, indicating a drop in hunting in unit 4 and the absence of domesticated cereals and fauna in unit 5. Thus, unit 5 still depends on a hunting-gathering economy, while unit 4 contains remains of a food producing economy. This drastic cultural change happened in less than a century, and undoubtedly shows connections with the Fertile Crescent where the domesticated species come from [12]. However, as no colonies with similar cultural features to those known at that time in the Near East has been found in the Caucasus, the local communities undoubtedly took a great part in this Neolithisation process, as also pointed out by the regional variability at work from the very beginning of this Shomu-Shulaveri culture (see description below), also called the Aratashen-Shomu-Shulaveri Cultural Complex. Relations with the eastern wing of the Fertile Crescent seem to have existed already before the appearance of Neolithic features and this could explain the swiftness of the process, probably also made possible by the 8.2 ka climatic event which caused severe changes in the Fertile Crescent and in the Black Sea area as well.

Kalavan 1 burial ground, Armenia
Excavated as a rescue site by a French-Armenian team directed by C. Montoya. F. Le Mort excavated the Bronze Age burials.

This open-air site is situated in the Aragunyats Range on the north of Lake Sevan, Gegharkunik Province. It dates to the upper Paleolithic period (ca. 15,000-14,000 cal. BC) and was re-used as a burial place during the Early Bronze Age [13]. Five burials have been excavated and radiocarbon dated to ca. the middle of the 3rd millennium BC (from 2761 to 2456 cal. BC). They consist of pits covered with a small stone cluster except for one tomb without it. One grave contained three individuals but their bones were scattered. Most of the graves were provided with pottery related more to that known in the Kura Valley in Azerbaijan than to that known in other parts of Armenia. One child burial contained stone beads and dog's molars pendants, and one grave provided a metal bracelet and a metal ring. The most conspicuous aspect of these graves is that they present evidence of bone removals made after the inhumation, a practice also recorded in the Kura Valley at Mentesh Tepe (see [14] vol. 1, 252-285).

Two individuals from the Early Bronze Age provided DNA for analysis in [8].

Kamiltepe, Azerbaijan
Excavated by a German-Azerbaijanese team directed by B. Helwing (DAI, Berlin) and T. Aliyev (Institute of Archaeology, Academy of Sciences, Baku) between 2009 and 2015 with funds from the DAI and the German-French ANR Ancient Kura and Kura in Motion.

The site is located in the Mil Plain, in the area of Agjabedi close to the foothills of the Qarabagh. It was in the process of destruction. Its main occupation dates to the Neolithic period (ca. 5500 BCE) and was re-used for burials much later, during the Iron Age. It consists of a monumental circular mudbrick platform partly surrounded by circular and rectangular constructions with evidence of storage. Up to now its plan is unique. It possibly served as a support for light wattle and daub shelters and there is also evidence of food preparation on a large scale. Many repairs and reconstructions are attested [15,16]. At the time of excavations, the area was not yet irrigated with modern canals and a large survey around the site led to the discovery of numerous other Neolithic and a few Chalcolithic sites [17], some of which have been partly excavated. The Neolithic sites have various architectures, from semi-subterranean round buildings surrounded with ditches to rectangular ones. Most are very low mounds of rather short duration. Altogether, the work done has brought to better light a branch of the Neolithic of Southeastern Azerbaijan partly known also in the Mughan area (see below Polutepe), in the Qarabagh plain [18] and in Nakhchivan (see below Kültepe) albeit with probable

regional differences. It is different from the Shomu-Shulaveri Culture by its pottery, mainly vegetal-tempered, and by the appearance of painted decoration after a phase of monochrome and sometimes red-slipped ware. The two areas were nevertheless in relation one with the other. Importantly, the painted ware is seen as related with that from Northwest Iran. From the 23 radiocarbon analyses, it dates between ca. 5600 and 5300 BCE.

No ancient DNA analyses have still been published for this site.

Kaps, Armenia
Excavated by L. Petrosyan (Institute of Archaeology and Ethnology, Yerevan) and by L. Yeganyan and H. Khachatryan (Sirak Museum).

Situated in the Sirak Province on the left bank of the Akhuryan river, the graves were excavated as a rescue project. The tombs of Kaps are located on the southern and eastern slopes of a ridge on the left bank of the Akhuryan River. Out of four stone-chamber tombs that were discovered, only one (2 x 2 x 1m) with a double-burial had remained untouched. The entrance of the chamber was located in the eastern wall. There are three synchronous burials in this chamber. Two human skeletons lying on their right side were found directly beside the western wall, whereas the third burial was documented behind the immobile entrance stone. The head of the latter skeleton was oriented towards to the east. Early Bronze Age Red-Black-ceramic vessels and bronze objects were found in these chambers. A synchronous settlement with portable fire-installations and ceramics was documented near the burial ground. However, the settlement had been damaged during construction works. The archaeological site of Kaps is dated to the second stage of the KA culture, though two radiocarbon dates indicate rather its early phase, i.e. the second half of the 4th millennium.

From [19], two individuals associated with the Kura-Araxes culture produced genome-wide data:

- ARM001.A0101.TF1: 3501-3128 calBCE (4595±30BP), 3631-3369 calBCE (4695±40BP, KIA44691)
- ARM002.A0101.TF1: 3338-3030 calBCE (4475±30BP, KIA44692), 3341-3030 calBCE (4480±30BP) 3366-3106 calBCE (4545±25BP, KIA44693)

Kmlo-2, Armenia
Excavated by a French-Armenian team under the direction of M. Arimura.

Cave situated east of the Aragats massif, at 1700m asl. A medieval occupation has disturbed the lower levels containing evidences of five occupation layers dated to the 12th-11th, mid 9th-mid 8th and end of 6th-5th millennia BC [20,21]. Hearths, many obsidian tools and animal bones constitute the main finds but from the thin deposits, the cave was used only as a temporary hunting camp. The tools were produced on the spot on the locally available obsidian. They include 30% of microliths. One of the most interesting finds is an obsidian artifact with continuous and parallel retouch on one or both edges executed by pressure flaking technique and called the "Kmlo tool" the function of which is unclear. It is said to be close to the "Çayönü tool" of southeastern Turkey dated to the PPNB (8th-7th millennia BC) though different in techno-morphological design, and is also similar to a "hooked tool" found in Georgia within the Paluri-Nagutni culture.

Kotias Klde, Georgia
Re-excavated in 2003-2005 by T. Meshveliani, G. Bar-Oz, O. Bar-Yosef et al.

Cave site situated in the Kvirila River basin of western Georgia. The deposits are divided into four layers, ranging from the Upper Palaeolithic to the Bronze Age. Layer B ('Mesolithic') is dated to the eleventh–ninth millennia BC and Layer A2 ('Early Neolithic') to the eighth millennium BC (7690–7300 cal BC) [21,22]. The site produced rich lithic assemblages and exclusively faunal wild species, mainly wild boar and bear. The lithic artefacts are mainly made from flint/radiolarite, a local raw material. A few obsidian pieces are also present, indicating long-distance expeditions or trade for the acquisition of this material as the nearest source is Chikiani, some 80 km from the site.

The Mesolithic industry of Kotias Klde is characterised by microliths. Backed bladelets including broken pieces are quite numerous, which may show a continuous tradition from the late Upper Palaeolithic. A significant Mesolithic tool type is the scalene triangle (backed bladelet with obliquely truncated ends). End scrapers made on flakes and blades are dominant among retouched tools, while burins are less common. In the 'Neolithic' layer, tools with hooked projections are similar to those found in the Paluri-Nagutni sites. According to the excavators, the Mesolithic and Neolithic materials have close parallels with the assemblages of Layers V and IV at the nearby site of Darkveti.

One individual dated around 7940-7600 calBCE is published by [23].

Kültepe, Nakhchevan, Azerbaijan
Excavated by O.A. Abibullaev 1951-1964. Recently re-opened by a French-Azerbaijanese team directed by C. Marro (CNRS, Lyon) and V. Bakhshaliyev (Institute of Archaeology, Nakhchevan-Baku).

The site is located in a key position between N.W. Iran and the Lesser Caucasus, along a left side tributary of the Araxes River. It was partly buried under alluvium and had been damaged by modern activity so that its original size (over 1.5ha) is unknown. It is the first one to have documented Neolithic levels (at that time said to be Chalcolithic) on over 9m high in the southern Caucasus. Other periods were present, including a Kura-Araxes one, but they have been leveled up. Several large circular structures, a few rectangular ones and many hearths were then discovered at different levels. A great number of graves (85) also come from these levels, with 1 to 4 individuals from infants to adults in flexed position. Some were void of inventory, but most were provided with various artifacts, from tools including a sickle from a deer antler; obsidian blades and one nucleus; shell-, unknown stone-, turquoise-, carnelian-, bone- and one copper- beads; pendant from a tooth's wild animal; painted or unpainted pottery; a stone mass-head, and in one of them a dog was also found. Moreover, the discovery of painted pots in the Halaf tradition established with certainty a link between the southern Caucasus and Northern Mesopotamia [24].

The recent excavations [25] have provided 34 radiocarbon dates: thirty-one place the main occupation between ca. 6200 and 5000 cal. BC, which would make this site the earliest one occupied during the Neolithic period in Southern Caucasus; one analysis points at a reoccupation between ca. 4500-4350 cal. BC and is complemented by the find of a grave and potsherds attributed to the beginning of the 4th millennium; two other analyses show a resettlement during the last third of the 4th millennium and are confirmed by a grave with three individuals and several potsherds of the Kura-Araxes period. The Neolithic occupation is divided into two levels. Level 1 (extreme dates from 6372 to 5626 cal. BCE) has been excavated on a very small area (12,5m2) and has no evidence of mud-built architecture but only that of postholes; cereals and fauna are already fully domesticated and about 2000 potsherds, mostly vegetal-tempered and sometimes cream-slipped and burnished, are already present among which two crudely painted sherds. This abundance in pottery is totally unusual for such early levels in the rest of the Southern Caucasus. In level 2 (extreme dates from 5987 to 4840 cal. BC), circular houses (made of mud or mud lumps) are present, the pottery is not as well finished as in level 1 but is sometimes provided with lugs or with an applied motif or band, while painted pottery is totally absent. Shapes, color and temper in general relate the assemblage with that of the Mil and Mughan plains (see here Kamiltepe and Polutepe). Most of the lithic industry in both levels

is made of obsidian and only a small part is made of flint. The authors notice that level 1 did not provide any microliths that could have related the initial occupation to the previous Mesolithic tradition. This, together with the exiguity of the excavations into what is left of the original mound, as well as the oddity of such an abundance of pottery in the very beginning of the occupation and the overlap in the dates between Levels 1 and 2, leave doubts as to part of the finds.

No ancient DNA analysis has still been published for this site.

Masis Blur, Armenia

See below at Shomu-Shulaveri Culture

Mentesh Tepe, Azerbaijan

Excavated between 2008 and 2015 by a French-Azerbaijanese team under the direction of Bertille Lyonnet (CNRS, Paris) and Farhad Guliyev (Institute of Archaeology, Academy of Sciences, Baku).

Mentesh Tepe (40.9418889_N, 45.8327778_E) is a small mound on the lower fan of the Zeyem Chaj – a left bank tributary of the Kura River. Its remains probably covered at least ca. 0.5 ha but it was totally scalped recently and partly lays also beneath modern houses. Remains of its lower/main occupations were fortunately preserved under the surface. Four main periods interrupted by gaps of more or less long duration have been identified. 1/ The earliest (period I) is dated by 21 radiocarbon dates between ca. 5993-5537 BC [26]. It is related to the Late Neolithic Shomu-Shulaveri Culture (SSC) with circular architecture either above ground or partly dug into it, but, being on the most eastern edge of the SSC, it also presents some specific features especially in the pottery, and relations with areas further east in the Mil'-Karabagh Steppe (Kamiltepe) have been underlined [26]. This Neolithic period I provided several infant burials and an exceptional collective grave most probably dug into an abandoned circular house with 30 individuals of mixed ages and sexes in primary position, with no evidence of trauma, enamel hypoplasia or other pathology indicating a violent episode or starvation. In this collective grave, the imbrication of some of the skeletons tend to point at simultaneous inhumations, while a layer of sediment covering others indicates a possible lapse of time between them. The good bone preservation and their excavation by a group of anthropologists provided many details. They show an artificial distribution of sexes (more women than men) and ages (no infant less than one year, many immatures (65%) [27]. 2/ After a long abandonment of about 1000 years, a very short reoccupation with a post-holes light architecture made probably by mobile groups is dated to ca. 4600 BCE (period II). 3/ It was followed three centuries later by an important settlement (period III, ca. 4350-4100 BCE) with a totally new rectangular, and possibly tripartite Mesopotamian-like architecture. Copper-based metallurgy at that time shows a quick development. This period at Mentesh clearly announces the further development and tighter relations that are obvious in the first half of the 4th millennium BCE between Southern Caucasus (Leilatepe culture), Northern Caucasus (Maikop culture) and Northern Mesopotamia (LC2-3) [7]. 4/ After another abandonment of long duration (ca. 600 years), the tepe already probably looking like a natural hill was re-used for burials (period IV). A first kurgan (Kurgan 4) was built for collective/successive inhumations (at least 39 individuals) and used during the early phase of the Kura-Araxes culture in the second half of the 3rd millennium BC. The kurgan was put to ritual fire at the end, leaving the human bones in a very bad state of preservation. The site was possibly short-term occupied after that, until a second kurgan (Kurgan 54) was built ca. 2500-2400 BCE, containing three individuals and a four-wheel cart. The rather rich material found with them – gold and carnelian beads and ring, an imported shell ring, hair-spirals and bracelets made of tin-bronze, a silver small casket and a good amount of pottery – relate it to the Martkopi phase of the so-called Early Kurgan Culture [28], a period when long distance connections start to develop.

One individual (Individual # 1), an immature aged between 10 and 14 years from the Late Neolithic collective burial has been previously analyzed which produced genome-wide data and was included in the genetic analyses published by [3].

Dating of human bone: 5993-5784 cal. BCE (7010 ± 45 BP, Sac A 41508/Gif-13016); dating of human tooth: 5717-5670 cal BCE (6802 ± 23 BP, MAMS-40333)

Polutepe, Azerbaijan

Excavated by the Mughan Neolitic-Eneolitic expedition of the Institute of Archaeology and Ethnography of Azerbaijan National Academy of Sciences between 2006 and 2017, under the direction of Tufan Akhundov.

The site of Polutepe (39.5186111_N, 48.6500000_E) is situated on the south bank of the Injachay River, on the territory of the village of Uchtepe, district of Jalilabad, Azerbaijan. The area represents a narrow band in the eastern part of the Mughan steppe limited by the spurs of the Brovary Range to the west and the Caspian Sea to the east. Presently, the settlement looks like a 6 ha ashy hill up to 6m high. Its central part is occupied by the modern cemetery of the Uchtepe village. Extensive excavations have revealed 7m of cultural layers [29]. The 1m high upper layer represents the remains of a IX-XIth centuries CE Medieval settlement, with simple and glazed ceramics characteristic of that time. In between these Medieval and the earlier Neolithic layers, pottery of the Kura-Araxes culture and of different stages of the Middle Bronze Age have been found. The lower 6 m layers of cultural deposits belong to the Neolithic period, more specifically to what the author has defined as the "Mughan Neolithic" culture. A large number of pottery sherds, bone and stone tools and other items, several burials, remains of different mudbrick constructions, and pottery kilns were revealed in the different construction levels of this thick layer. The greatest part of the excavated area concerns a productive sector of the settlement. The constructions present different plans, either round, oval or rectangular. The burials include individuals of both sexes and all age groups, from infants to old adults. The graves consist in shallow pits made on different plots among the constructions. The deceased were placed in a crouched position with different degrees of flexion. They were often covered with red ochre and provided with beads and a pottery bowl. The lower horizons of the cultural layers revealed a hearth for cult and over two dozen small stylized female clay figurines.

One individual from Polutepe, POT002 (Polutepe Burial N2) had a tooth dated to 5508-5376 cal BCE (6491 ± 26 BP, MAMS-40331) and was analyzed for aDNA analyses by [3].

Shomu-Shulaveri Culture (also called Aratashen-Shulaveri-Shomu Cultural Complex), South Caucasus

First identified in the 1960s at the site of Shomutepe in the outskirts of Agstafa, as well as at many other sites in the Qazakh, Agstafa and Tovuz districts of Western Azerbaijan (synthesis in[30]), this culture was soon after also discovered in the contiguous area of Marneuli, Georgia at Shulaveris Gora and a number of sites around (synthesis in [31]). Due to its already well-developed features in the architecture, or in the domestication of plants and animals, and due to the absence of radiocarbon dates at that time, it was attributed to the Chalcolithic period. Research made from the 2000's in different areas of the South Caucasus, either on sites already partially excavated (in Georgia, Aruchlo[32], Gadachrili Gora [33,34], Shulaveris Gora), in Armenia (Masis Blur[35]) or on new ones (in Azerbaijan, Göy Tepe[36], Hacı Elamxanlı Tepe[37], Kiçik Tepe[38], Mentesh Tepe[39] (for this site see more information above)) including in Armenia in the Ararat Plain (Aratashen[40], Aknashen[41]), have corrected its chronological attribution now well established in the 6th millennium, with the earliest sites (Aknashen, Armenia, and Haci Elamxanli Tepe, Azerbaijan) starting at the very beginning of this millennium, and the latest occupations attested up to ca. 5300 BCE, for instance at Aruchlo, Georgia. The extreme speed with which this culture spread as shown by the succession from the Mesolithic to the Neolithic levels at Damjili Cave (see the description of this site above), together with the presence of a small bunch of foreign pottery related to the Samarra and Halaf cultures in the earliest levels (at Aratashen, Aknashen, Masis Blur and Haci Elamxanli) and that of genetically non-local domesticated plants and

animals, point to an exterior intrusion of groups from SE Anatolia/Northern Mesopotamia into the area.

All the sites are small (ca. 1ha), mound-like (up to 10-12m high) with a rather quick succession of construction levels pointing at the short duration of the houses. Though these sites share many features in common (circular architecture, similar bone and lithic industries, same cultivated and raised species, similar experiments in copper production, etc.), justifying their integration into a common cultural complex, regional and chronological specificities are also present, especially in the pottery (with different tempers, shapes and decoration according to the area and the time-period), the overall shape of the constructions (first "snowman"-like, then included in a courtyard), that of the building techniques ("bauge", flat or plano-convex bricks), the presence of ditches or not, etc. They differ from the contemporary Neolithic sites found more to the East in Nakhchivan (Kültepe 1) or in the Mil and Mughan Steppes (Polutepe, Kamiltepe, etc.) though we still lack of large excavations and detailed publications from this eastern area to have a clear view of its material culture. Moreover, interactions between the two groups are visible for instance at Mentesh Tepe, the easternmost of the Shomu group, or at Kültepe with the presence there too of Halaf pottery. Burials are not always present and do not seem to follow a homogeneous scheme: at Aruchlo, one case of cremation has been found; at Aknashen and Masis Blur respectively 5 and 3 individual burials were discovered; at Mentesh Tepe, besides 3 immature or infant burials, an atypical burial pit in an abandoned house contained 30 skeletons (mainly women and young children) all buried within a short time span probably as a result of a sudden episode, possibly a fire, while no Neolithic burials have been found at Aratashen, Gadachrili Gora, Haci Elamxanli, Göy or Kiçik Tepe.

One child from Mentesh Tepe (MTT1 had a tooth dated to 5717-5670 cal BCE (6802 ± 23 BP, MAMS-40333) and was analyzed for aDNA analyses by [3].
One newborn from Aknashen (I3931), dated to 5720-5564 cal. BCE (6730 ± 40 BP, Poz-70153), was analyzed for a DNA analysis by [42] where it is mentioned erroneously as 5985-5836 cal. BCE.
One male individual from Masis Blur (I3930), dated on a tooth to 5633-5532 cal. BCE (6665 ± 25 BP, PSUAMS-3057) was analyzed for a DNA analysis by [42].

Soyuq Bulaq, Azerbaijan
Excavated by N. Museibli (Institute of Archaeology and Ethnology, Baku) and by a French-Azerbaijanese team directed by B. Lyonnet (CNRS, Paris) and T. Akhundov (Institute of Archaeology and Ethnology, Baku).

This cemetery of kurgans is situated in the Agstafa district in western Azerbaijan, close to the Georgian border on the left bank of the Kura River. It consists of very low kurgan-mounds surrounded by a circle of large river pebbles and dates to the first half of the 4th millennium. Most of the kurgans have been investigated as rescue excavations during the construction of the BTC pipe-line in 2005 (Museibli 2014). In 2006, a French-Azerbaijanese team directed by B. Lyonnet (CNRS, Paris) and T. Akhundov (Institute of Archaeology, Academy of Sciences, Baku) excavated some of the kurgans outside the pipeline working area [43]. Some of them contained in the center a rectangular pit-grave surrounded by a mudbrick wall. One was richly furnished with a copper knife, a stone scepter in the shape of an equid, one lapis bead (the earliest found in the South Caucasus in a known context) and several carnelian and paste beads, as well as several gold and silver-copper beads. The human bones recovered in this grave were badly preserved (radiocarbon dated from a tooth: 3951-3759 cal BCE, UB-7609) and, due to their high position in the grave may have belong to a woman positioned above the supposed man's original grave at the bottom but where no bones were recovered. This grave was accompanied by a "fake" grave packed with pebbles arranged immediately under the circle on the surface and containing over 80 shell beads and one in black steatite but no human bones. Among the kurgans excavated, several did not show any trace of inhumation and most of those with graves contained only parts of human skeletons leading to propose a funerary ritual with a possible exposition of the corpses before internment. This proposition seems to find correlates

in the funerary ritual from Areni-1 where some bones wear traces of dogs' teeth (see [5]). The Soyuq Bulaq kurgans are the earliest known in Southern Caucasus together with that of Kavtiskhevi in Georgia [44] and those – possibly even slightly earlier - of Aknalitch in Armenia [45]. They are clearly related on the one hand to the kurgans of Sé Girdan on the south of Lake Urmia ([46,47] with corrections on the dates in [48]) and, on the other hand, to those of the Maikop culture in the North Caucasus, as well as to the Leilatepe culture [43]. The two radiocarbon dates from UB place the Soyuq Bulaq cemetery between ca. 3950 and 3650 BC.

Unfortunately, the material and the bones from the French-Azerbaijanese excavations could not be recovered recently in the funds of the Institute of Archaeology in Baku.

No ancient DNA analyses have still been published for this site.

Talin necropolis, Armenia
Excavated by G. Sargsyan and P. Avetisyan and F. Muradyan (Institute of Archaeology and Ethnography, Erivan).

The necropolis is situated outside the actual city of Talin (Aragats Province). It covers different periods from the Early Bronze Age (Kura-Araxes culture) in last quarter of the 4th millennium BC to the Hellenistic period [49]. The Early Bronze Age is represented by four tombs under tumuli ca. half a meter high, some with a grave pit, others with an above-ground grave, and one is surrounded with a cromlech. Tomb 12 contained one individual while the other tombs had five to nine individuals. Two radiocarbon dates place them between ca. 3300-2900 BC. An enclosure in two parts, one paved and the other not (tramped surface) is surrounded by a stone wall, and also covered by a tumulus containing many sherds, animal bones and obsidian fragments. This enclosure is said to be for rituals and is also related to this period. The tombs as the ritual place provided many finds, including pottery (part of which is decorated with geometric incisions), metal items (one ring with > 10% tin), faience and shell artifacts.

One individual from Talin tomb 115 is published in [8]: TA3/R8, 3347-3092 cal. BCE (4492 ± 29BP, OxA-31874)

Unakozovskaya, Russia
Excavated by N.G. Lovpache, the Adygey Pedagogical University, 1985-90.

Located in the Northwestern part of the Greater Caucasus close to the Klady cemetery (N 44.255876°, E 40.201139°), the Unakozovskaya caves belong to a series of Chalcolithic sites that have been found either in caves or below rock shelters, or as opened settlements in the valleys of the Western Greater Caucasus rivers, both on the northern side (like Meshoko in Adygej, Zamok in Stavropol' Kraj) and on the southern side (Darkveti in Imereti, Georgia). This has led to identify this period as that of the Darkveti-Meshoko or Zamok/Meshoko horizon [50]. This pre-Maikop culture is now dated from ca. 4700 to 4000 BC by radiocarbon dates. The pottery is often decorated with incised and applied ornaments, including pearls pushed from inside the pots. Polished stone bracelets are also one of the typical artefacts related to this culture. Long considered as a component of the Maikop culture, it is now clear that it is different and precedes it [7]. Besides the sites mentioned above, Svobodnoe is a fortified settlement at the northern foothills of the Greater Caucasus with black polished pottery, but there is still uncertainty on its affiliation to this horizon. In the northwest Caucasus, the environment today is densely forested, but it was characterized by a very different vegetation cover with open landscapes and much less forest until the end of the Middle Bronze Age (ca. 3500 cal BP).

Three *Darkveti-Meshoko Eneolithic* children produced genome-wide data in [19]:

• I2055, skeleton 2a: 4680-4486 cal BCE (5718±29BP, OxA-43740)
• I2056, skeleton 2b: 4599-4456 cal BCE (5687±30BP, OxA-43741)
• I1722, skeleton 3: 4536-4371 cal BCE (5635±27BP, OxA-43742)

Velikent, Daghestan
Excavated by different actors during Soviet times, and by a Daghestan-American Expedition in the 1990s directed by R.G. Magomedov (Institute of History, Archaeology and Ethnography, Makhatchkala) and P.L. Kohl (Wellesley College, USA).

The site (N 42.179802°, E 48.066089°) is located on the corridor plain along the west coast of the Caspian Sea relating the southern Caucasus and Iran to the northern Eurasian Steppe. It includes several mounds, two of them being settlements while three are cemeteries with 15 collective catacomb tombs [51,52]. All are dated between ca. 3500-2000 BC by over 20 radiocarbon dates. The settlements are partly disturbed, but less than the cemeteries that were largely destroyed by modern activities and subject to rescue excavations. The settlements were occupied successively, mound II, the best investigated, being older (mostly second half of the 4th millennium) than mound I (second half of the 3rd millennium). Most of the constructions were dug-in structures (mainly circular pit-houses), except for the oldest circular structure in mound II that was built with mudbricks above ground. The cemeteries consist mainly in large catacomb structures with collective inhumations containing up to 600 individuals, and the inventory in some of them is especially rich in pottery and metal tools, weapons and ornaments part of which is made of tin bronze. The metallurgical skills of the inhabitants have been highlighted since the beginnings of the occupation. The three radiocarbon dates from two of the catacomb graves point to their functioning between ca. 2650 to 2000 BC. The overall material culture presents specific features widespread not only along the coast into actual Azerbaijan but also within the mountainous area up to Chechnya that distinguishes it as a variant from the contemporary Kura-Araxes culture. The catacomb graves as for them are contemporary to the Martkopi and Bedeni phases of the Early Kurgan culture in southern Caucasus.

Two individuals not radiocarbon dated produced genome-wide data published in [19]:
• VEK006.A0101.TF (BZNK-486/5): relative date 3000-2800 BCE
• VEK007.A0101.TF + VEK009.A0101.TF (BZNK-486/6;8): relative date 3000-2800 BCE; 2nd degree relative of VEK006

Supplementary References

1. Akhundov, T. The South Caucasus on the threshold of the Metal Age. in Context and connection : studies on the archaeology of the ancient Near East in honour of Antonio Sagona. (eds. Batmaz, A., Bedianashvili, G., Michalewicz, A. & Robinson, A.) 49–76 (Orientalia Lovaniensia Analecta 268., 2018).

2. Akhundov, T. Alkhantepe Yaşayiş yeri. Leylatepe enenesi. (2021).
3. Skourtanioti, E. et al. Genomic History of Neolithic to Bronze Age Anatolia, Northern Levant, and Southern Caucasus. Cell 181, 1158-1175.e28 (2020).
4. Wilkinson, K. N. et al. Areni-1 Cave, Armenia: A Chalcolithic–Early Bronze Age settlement and ritual site in the southern Caucasus. J. F. Archaeol. 37, 20–33 (2012).
5. Areshian, G. E. et al. The chalcolithic of the Near East and south-eastern Europe: discoveries and new perspectives from the cave complex Areni-1, Armenia. Antiquity 86, 115–130 (2012).
6. Lyonnet, B. La Mésopotamie et le Caucase du Nord au IVe et au début du IIIe millénaires av. n.è. : leurs rapports et les problèmes chronologiques de la culture de Majkop. État de la question et nouvelles propositions. in (2000).
7. Lyonnet, B. La culture de Maikop, la Transcaucasie, l'Anatolie orientale et le Proche-Orient : relations et chronologie. in Les cultures du Caucase (VIe- IIIe millénaires av. n. è.). Leurs relations avec le Proche-Orient. (ed. Lyonnet, B.) 132–161 (2007).
8. Lazaridis, I. et al. Genomic insights into the origin of farming in the ancient Near East. Nature 536, 419–424 (2016).
9. Armikhanov, K. A. Chokhskoe poselenie. Chelovek i ego kul'tura v mezolite i neolite Gornogo Dagestana. (1987).
10. Armikhanov, K. A. Khronologija kul'turnykh otlozhenij Chokhskogo mnogoslojnogo poselenija (po dannym na 2022 g.). Istor. Arkheologija i Etnogr. Kavk. 18, 715–728 (2022).
11. Nishiaki, Y. et al. The Mesolithic-Neolithic interface in the Southern Caucasus: 2016–2017 excavations at Damjili Cave, West Azerbaijan. Archaeol. Res. Asia 19, 100140 (2019).
12. Kadowaki, S., Ohnishi, K., Arai, S., Guliyev, F. & Nishiaki, Y. Mitochondrial DNA Analysis of Ancient Domestic Goats in the Southern Caucasus: A Preliminary Result from Neolithic Settlements at Göytepe and Hacı Elamxanlı Tepe. Int. J. Osteoarchaeol. 27, 245–260 (2017).
13. Montoya, C. et al. The Upper Palaeolithic site of Kalavan 1 (Armenia): An Epigravettian settlement in the Lesser Caucasus. J. Hum. Evol. 65, 621–640 (2013).
14. Poulmarc'h, M. Pratiques funéraires et identité biologique des populations du Sud Caucase, du Néolithique à la fin de la culture Kura-Araxe (6e-3e millénaire av. J.-C.): une approche archéo-anthropologique. (Université Lumière Lyon 2., 2014).
15. Helwing, B. & Aliyev, T. Field Work in the Mil Plain: the 2010-2011 Expedition. in Ancient Kura 2010-2011: The first two seasons of joint field work in the Southern Caucasus. (eds. Lyonnet, B. et al.) 4–17 (2012).
16. Helwing, B. & Aliyev, T. Excavations in the Mil Plain Sites, 2012-2014. in (eds. Helwing, B. et al.) 11–42 (Dietrich Reimer, 2017).
17. Ricci, A. Archaeological Landscape Studies within the 'Kura in Motion' Project: the 2012-2014 Field Activities. in The Kura Projects. New Research on the Later Prehistory of the Southern Caucasus (eds. Helwing, B. et al.) 305–323 (Dietrich Reimer, 2017).
18. Almammedov, K. Qarabağin arxeoloji abideleri toplusu (The corpus of archaeological monuments of Garabagh). (2016).
19. Wang, C.-C. et al. Ancient human genome-wide data from a 3000-year interval in the Caucasus corresponds with eco-geographic regions. Nat. Commun. 10, 590 (2019).
20. Arimura, M., Badalyan, R., Gasparyan, B. & C., C. Current neolithic research in Armenia. Neo-Lithic 1, 77–85 (2010).
21. Chataigner, C., Badalyan, R. & Arimura, M. The Neolithic of the Caucasus. in Online Only -- Archaeology (Oxford University Press, 2014). doi:10.1093/oxfordhb/9780199935413.013.13

22. Meshveliani, T. et al. Mesolithic Hunters at Kotias Klde, Western Georgia: Preliminary Results. Paléorient 33, 47–58 (2007).
23. Jones, E. R. et al. Upper Palaeolithic genomes reveal deep roots of modern Eurasians. Nat. Commun. 6, 8912 (2015).
24. Abibullaev, O. A. Eneolit i Bronza na territorii Nakhichevanskoj ASSR. (1982).
25. Marro, C., Bakhshaliyev, V., Berthon, R. & Thomalsky, J. New light on the Late Prehistory of the South Caucasus. Paléorient 45, 81–114 (2019).
26. B, L. & Guliyev, F. Mentesh Tepe (Azerbaijan). Preliminary report on the 2012-2014 excavations. in The Kura Projects. New Research on the Later Prehistory of the Southern Caucasus (eds. Helwing, B. et al.) (Dietrich Reimer, 2017).
27. Pecqueur, L. & Jovenet, E. La sépulture 342 de Mentesh Tepe (Azerbaijan) : un exemple de chaîne opératoire funéraire complexe au Néolithique. Etude préliminaire. in The Kura Projects. New Research on the Later Prehistory of the Southern Caucasus. (eds. Helwing, B. et al.) 163–178 (Dietrich Reimer, 2017).
28. Pecqueur, L., Decaix, A. & Lyonnet, B. Un kourgane de la phase Martkopi à Mentesh Tepe (période des Premiers Kourganes, Bronze Ancien). in The Kura Projects. New Research on the Later Prehistory of the Southern Caucasus. (eds. Helwing, B. et al.) 179–192 (Dietrich Reimer, 2017).
29. Akhundov, T. Archaeological Sites of the Mugan Steppe and Prerequisites for Agricultural Settlement in the South Caucasus in the Neolithic-Eneolithic. Strat. Plus 2, 219–236 (2011).
30. Narimanov, I. G. Drevnejshego zemledel'chesko-skotovodcheskogo naselenija Azerbajdzhana/The oldest farmers and pastoralists of Azerbaijan. (1987).
31. Kushnareva, K. K., Kušnareva, K. C. & Michael, H. N. The Southern Caucasus in Prehistory: Stages of Cultural and Socioeconomic Development from the Eighth to the Second Millennium B.C. (University of Pennsylvania Press, Incorporated, 1997).
32. Hansen, S. & Mirstkhulava, G. Excavations at Aruchlo 2005-2014. in The Kura Projects New Research on the Later Prehistory of the Southern Caucasus. (eds. Helwing, B. et al.) 195–200 (Dietrich Reimer, 2017).
33. Batiuk, S. et al. The 2017-218 Gadachrili Gora Regional Archaeological Project (GRAPE): Second Preliminary Report. Anatolica XLV, 43–76 (2019).
34. Hamon, C. et al. Gadachrili Gora: Architecture and organisation of a Neolithic settlement in the middle Kura Valley (6th millennium BC, Georgia). Quat. Int. 395, 154–169 (2016).
35. Martirosyan-Olshansky, K. Masis Blur: A Neolithic Settlement in the Ararat Plain, Armenia. (2014).
36. Watkins, T. Yoshihiro Nishiaki and Farhad Guliyev (eds.) – Göytepe: Neolithic Excavations in the Middle Kura Valley, Azerbaijan. Paléorient 127–129 (2021). doi:10.4000/paleorient.1437
37. Nishiaki, Y., Guliyev, F. & Kadowaki, S. Hacı Elamxanlı Tepe. The Archaeological Investigations of an Early Neolithic Settlement in West Azerbaijan. (2021).
38. Palumbi, G. et al. New data and perspectives on the early stages of the Neolithic in the Middle Kura River Valley (South Caucasus). The 2017–2019 excavations at Kiçik Tepe, Western Azerbaijan. Archaeol. Res. Asia 27, 100308 (2021).
39. Lyonnet, B. et al. Mentesh Tepe, an early settlement of the Shomu-Shulaveri Culture in Azerbaijan. Quat. Int. 395, 170–183 (2016).
40. Badalyan, R. et al. New data on the Late Prehistory of the Southern Caucasus. The excavations at Aratashen (Armenia): preliminary report. in Les cultures du Caucase (VIe- IIIe millénaires av. n. è.). Leurs relations avec le Proche-Orient (ed. Lyonnet, B.) 37–61 (2007).
41. Badalyan, R., Chataigner, C. & Harutyunyan, A. The Neolithic Settlement of Aknashen (Ararat Valley, Armenia), Excavation seasons 2004-2015. (2022).
42. Lazaridis, I. et al. Ancient DNA from Mesopotamia suggests distinct Pre-Pottery and Pottery Neolithic migrations into Anatolia. Science (80-.). 377, 982–987 (2022).
43. Lyonnet, B. et al. Late Chalcolithic Kurgans in Transcaucasia. The cemetery of Soyuq Bulaq (Azerbaijan). Archäoligische Mitteilungen aus Iran und Turan 40, 27–44 (2008).
44. Makharadze, Z. Nouvelles données sur le Chalcolithique en Géorgie orientale. in Les cultures du Caucase (VIe- IIIe millénaires av. n. è.). Leurs relations avec le Proche-Orient. (ed. Lyonnet, B.) 123–131 (2007).

45.Muradyan F. Discovery of the first Chalcolithic burial mounds in the Republic of Armenia. in Stone Age of Armenia (eds. Gasparyan, B. & Arimura, M.) 339–365 (2014).
46.Muscarella, O. W. The Tumuli at Sé Girdan: A Preliminary Report. Metrop. Museum J. 2, 5–25 (1969).
47.Muscarella, O. W. The tumuli at Sé Girdan: second report. Metrop. Museum J. 4, 5–28 (1971).
48.Muscarella, O. W. The chronology and culture of Sé Girdan: Phase III. Anc. Civilizations from Scythia to Sib. 9, 117–131 (2003).
49.Badalyan, R. & Avetisyan, P. S. Bronze and Early Iron Age Archaeological Sites in Armenia, I, Mt Aragats and its Surrounding Region. (2007).
50.Trifonov, V. Darkveti-Meshokovskaja kul'tura. in Tret'ja Kubanskaja Arkheologicheskaja Konferentsija 190–194 (2001).
51.Kohl, P. L. The Making of Bronze Age Eurasia. (Cambridge University Press, 2007). doi:10.1017/CBO9780511618468
52.Magomedov, R. & Kohl, P. Early Bronze developments on the West Caspian Coastal Plain. Paléorient 93–114 (2014).
