# Supplementary material for: Genome-wide analysis of a collective grave from Mentesh Tepe provides insight into the population structure of early neolithic population in the South Caucasus
Source: Commun Biol. 2023 Mar 25;6:319. doi: 10.1038/s42003-023-04681-w (PMC10039893; doi:10.1038/s42003-023-04681-w)
Supplement: Supplementary file 3 — Description of Additional Supplementary Files [file 42003_2023_4681_MOESM3_ESM.pdf]

Description of Additional Supplementary Files

File name: Supplementary Data 1

Description: List of the samples from the Early Neolithic structure S342, indicating their archaeological ID, C14 dates, their genetic sex, their mitochondrial and Y chromosome haplogroups and the quality of the genetic data. The informations not provided by this article come from 23,25..

File name: Supplementary Data 2

Description: Published ancient genome-wide data (modified .anno file from Allen Ancient DNA Resource (AADR); Skourtanioti et al. 2020)

File name: Supplementary Data 3

Description: All D-statistics used in this study, including the data behind Figure 2.c and Supplementary Figure 3 and 6

File name: Supplementary Data 4

Description: All qpAdm models calculated for this study, including the data behind Figure 2.d and Supplementary Figure 4 and 7.

File name: Supplementary Data 5

Description: Data from the READ analysis
